# A very rare case of incidental aortic valve fibrolipoma

**DOI:** 10.1093/icvts/ivab272

**Published:** 2021-10-12

**Authors:** Rickesh Bharat Karsan, Ronan Kelly, Estelle Healy, Reuben Jeganathan

**Affiliations:** 1 Department of Cardiothoracic Surgery, Royal Victoria Hospital, Belfast, UK; 2 Institute of Pathology, Royal Victoria Hospital, Belfast, UK

**Keywords:** Fibrolipoma, Cardiac tumours, Aortic valve repair

## Abstract

Cardiac valve fibrolipomas are extremely rare. We report a case of a 38-year-old female initially presenting with palpitations and moderate aortic incompetence who was found to have a lipomatous growth of the aortic valve. She underwent aortic valve repair with good postoperative results. Histopathogy verified the lesion as a fibrolipoma. This is the first reported case of fibrolipoma in the aortic valve, whilst aiming to consider repair as a surgical option in young patients with such growths.

## INTRODUCTION

Primary cardiac tumours are rare with a 0.5% incidence, of these 75% constitute benign pathologies including fibromas, fibroelastomas and haemangiomas [1]. Valvular tumours are especially rare, with fibroelastomas accounting for 75% of cases [2]. In such cases, patients can present with a myriad of symptoms including thromboembolic events, myocardial infarction, low cardiac output and arrhythmias. We report an extremely rare case of a symptomatic fibrolipoma of the aortic valve, which was successfully managed by the excision and repair of the native valve.

## CASE REPORT

**Figure 1:**
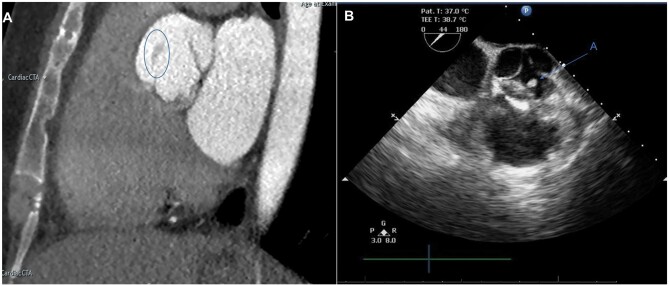
Gated CT of aortic valve. (**A**) Coronal view showing 7-mm lesion (blue outline). (**B**) Intra-operative transoesophageal echocardiogram. Short-axis parasternal view showing 7-mm ovoid lesion on right coronary cusp (**A**).

A 38-year-old female initially presented with palpitations, which were subsequently investigated highlighting no significant abnormality requiring immediate intervention. She was followed up with moderate aortic incompetence with and incidental finding of what was considered a likely fibroelastoma. She remained asymptomatic, EuroSCORE 3 (additive), 2.1 (logistic), EuroSCORE II 0.6. Her past medical history included costochondritis. Electrocardiogram showed to patient to be in normal sinus rhythm. A gated CT scan indicated a 7-mm ovoid mass on the right coronary cusp adjacent to the left ventricular outflow tract (Fig. 1A), which was also confirmed in intraoperative transoesophageal echocardiogram (Fig. 1B). There was no other valvular pathology noted. In view of her age, and risks posed by the lesion, she was considered for aortic valve surgery with a view this was to be a fibroelastoma.

Aortic valve surgery was performed via a mini-J shaped sternotomy into the right 4th intercostal space. Cardiopulmonary bypass was established with core cooling to 34°. Myocardial protection was achieved via antegrade cold blood cardioplegia. A hockey stick aortotomy was performed. A 7–8-mm tumour was visualized on the ventricular side of the right coronary cusp of a trileaflet aortic valve. The tumour was excised with a scalpel, leaving no injury or perforation. A saline test demonstrated good coaptation with no leaflet prolapse. The aortotomy was then closed in a double layer, the left ventricle de-aired and taken off CBP with no required inotropic support. No residual tumour was demonstrated in the postoperative transoesophageal echocardiogram with only trivial aortic incompetence present. We felt a repair was a much better option than replacement and would remove the risk of prosthesis related complications, including anti-coagulation. Her postoperative course was unremarkable with a good postoperative transthoracic echocardiogram (TTE) with mild regurgitation and no adverse issues at 6 week follow-up, further follow-up at 1 year showed no progression of aortic pathology.

**Figure 2:**
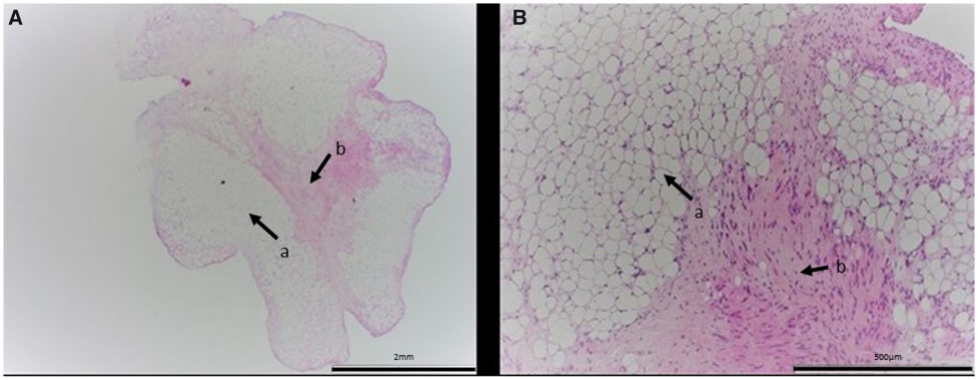
(**A**) Low power view (×40 magnification), haematoxylin and eosin-stained section showing a well-defined nodular lesion composed of mature adipocytes (a) with a central core of reactive collagen and myofibroblastic proliferation (b). (**B**) High power view (×400 magnification), haematoxylin and eosin-stained section showing mature adipocytes with a central core of reactive collagen and myofibroblastic proliferation.

Histopathology revealed a 7 mm × 3 mm × 1 mm well-defined nodular lesion with the bulk of the tissue composed of mature adipocytes, with a central core of reactive collagen and myofibroblastic proliferation contributing to the elaboration of the tumour (Fig. 2). Scattered lymphocytes were seen at the edges in keeping with a fibrolipoma. The tumour was considered benign.

## DISCUSSION AND CONCLUSION

Primary cardiac valve tumours are a rare but noted pathology and would normally result in the replacement of the affected valve. Lipomas themselves are rarer still with few reported cases [2]. Fibrolipomas are extremely rare with a literature search only identifying 2 documented cases in paediatric mitral valves [3, 4]. The case described is the first ever report of fibrolipoma of the aortic valve. The young age of the patient along with the well circumscribed nature of the tumour allowed for its excision with good post-operative echo, and would remove the risks and limitations associated with prosthesis related complications and life-long anticoagulation.

Fibrolipomas can present with clinical characteristics similar to those of classic lipomas of the aortic valve, including aortic insufficiency. Fibrolipomas are extremely rare, they are lipomas with focally increased fibrous tissue and have been noted in paediatric mitral valve pathologies as well as intrathoracic lesions [5]. Cardiac lipomas themselves do not pose problems; however, their size and site can result in various clinical manifestations. When growing on valves they can result in valve insufficiency and thromboembolism. Repair can be considered a safe option with good outcomes, where the lesion is well circumscribed and with thorough intraoperative assessment of the valve.

**Conflict of interest**: none declared.

## Reviewer information

Interactive CardioVascular and Thoracic Surgery thanks Andrew C. Chatzis, and the other, anonymous reviewer(s) for their contribution to the peer review process of this article.
